# A novel nomogram for predicting prolonged mechanical ventilation in lung transplantation patients using extracorporeal membrane oxygenation

**DOI:** 10.1038/s41598-024-62601-2

**Published:** 2024-05-22

**Authors:** Chenhao Xuan, Jingxiao Gu, Zhongping Xu, Jingyu Chen, Hongyang Xu

**Affiliations:** 1 The Affiliated Wuxi People’s Hospital of Nanjing Medical University, Wuxi People’s Hospital, Wuxi Medical Center, Nanjing Medical University, Wuxi, 214023 Jiangsu China; 2 Wuxi Lung Transplant Center, The Affiliated Wuxi People’s Hospital of Nanjing Medical University, Wuxi, 214023 Jiangsu China

**Keywords:** Prolonged mechanical ventilation, Lung transplant, Nomogram, Extracorporeal membrane oxygenation, Risk factors, Outcomes research, Cystic fibrosis

## Abstract

Prolonged mechanical ventilation (PMV) is commonly associated with increased post-operative complications and mortality. Nevertheless, the predictive factors of PMV after lung transplantation (LTx) using extracorporeal membrane oxygenation (ECMO) as a bridge remain unclear. The present study aimed to develop a novel nomogram for PMV prediction in patients using ECMO as a bridge to LTx. A total of 173 patients who used ECMO as a bridge following LTx from January 2022 to June 2023 were divided into the training (122) and validation sets (52). A mechanical ventilation density plot of patients after LTx was then performed. The training set was divided in two groups, namely PMV (95) and non-prolonged ventilation (NPMV) (27). For the survival analysis, the effect of PMV was assessed using the log-rank test. Univariate and multivariate logistic regression analyses were performed to assess factors associated with PMV. A risk nomogram was established based on the multivariate analysis, and model performance was further assessed in terms of calibration, discrimination, and clinical usefulness. Internal validation was additionally conducted. The difference in survival curves in PMV and NPMV groups was statistically significant (P < 0.001). The multivariate analysis and risk factors in the nomogram revealed four factors to be significantly associated with PMV, namely the body mass index (BMI), operation time, lactic acid at T0 (Lac), and driving pressure (DP) at T0. These four factors were used to develop a nomogram, with an area under the curve (AUC) of 0.852 and good calibration. After internal validation, AUC was 0.789 with good calibration. Furthermore, goodness-of-fit test and decision-curve analysis (DCA) indicated satisfactory performance in the training and internal validation sets. The proposed nomogram can reliably and accurately predict the risk of patients to develop PMV after LTx using ECMO as a bridge. Four modifiable factors including BMI, operation time, Lac, and DP were optimized, which may guide preventative measures and improve prognosis.

## Introduction

Lung transplantation (LTx) is an effective treatment for end-stage lung disease. Nowadays, thousands of LTxs are being performed worldwide on a yearly basis. Even though management and technology are constantly improving, the mortality rate is still higher than that of other solid organ transplants^1^. Prolonged mechanical ventilation (PMV) is an independent risk factor for survival after LTx^2^. The impact of PMV in LTx has been previously assessed retrospectively in the literature; the incidence of PMV was 29%, the post-operative complications of patients (primary graft dysfunction (PGD), infections, acute kidney injury, etc.) increased, the mortality rates of these patients were high, approximately 22% at 90-day and 44% at 1-year^3^. Due to the heterogeneity of LTx itself, the factors affecting PMV are complex, and they are divided into three aspects: pre-operative, intra-operative, and post-operative. Pre-operative primary disease leads to continuous deterioration of the health status, and intra-operative ischemia–reperfusion of the lung and surgical trauma, as well as the effects of post-operative rejection and infection affect the long-term prognosis^4^. Therefore, patients at high risk of PMV require identification to enable the guidance of preventive and interventional measures.

In recent years, extracorporeal membrane oxygenation (ECMO) has been widely used in LTx as a bridge, and has reduced the peri-operative mortality of critically ill patients during LTx^5^. Using ECMO as a bridge to LTx can achieve comparable survival to non-bridged patients^6,7^. The application of ECMO can improve the hemodynamic status or promote gas exchange and aid patients with severe PGD by supporting oxygenation and protecting transplant lung function. ECMO support has become an effective measure that ensures the safe transition of patients before surgery, reduces the risk of intra-operative surgery and anesthesia, and improves the prognosis of patients^8^. Even though LTx using ECMO as a bridge significantly increases duration of mechanical ventilation, few studies exist in the literature specifically for these patients. This study aimed to establish a nomogram for predicting PMV in patients using ECMO as a bridge to LTx.

## Methods

### Ethics

This retrospective study was conducted in accordance with the Declaration of Helsinki (as revised in 2013) and approved by the Ethics Commission of the Affiliated Wuxi People's Hospital of Nanjing Medical University (No. KY23161). The need for informed consent was waived by the Committee due to the retrospective nature of this study.

### Study design and patients

**Figure 1:**
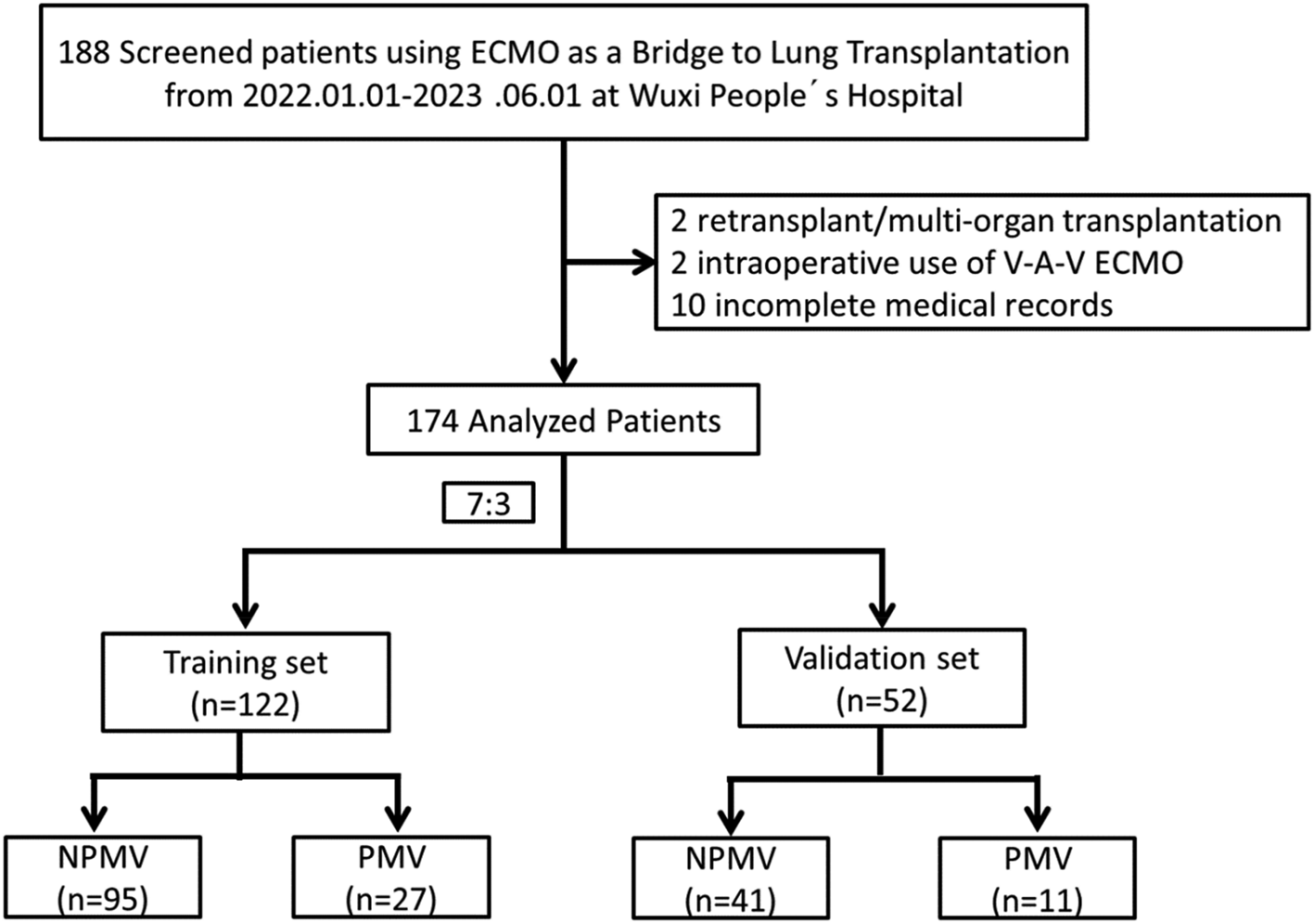
The flow diagram.

Between January 2022 and June 2023, the medical records of consecutive patients using ECMO as a bridge to LTx, with a 90-day survival follow-up were reviewed. Inclusion criteria: (1) Age older than 18 years; (2) Underwent LTx; (3) Postoperative survival time ≥ 5 days. Exclusion criteria: (1) Re-transplant/multi-organ transplantation; (2) Intra-operative use of V-A-V ECMO; (3) Incomplete medical records. All patients were randomly divided into training set and validation set at a ratio of 7:3. The training set was used to construct a nomogram for PMV and the validation set to conduct internal validation. The flow diagram is shown in Fig. 1.

### Data collection

The following data were collected from the hospital records: age, sex, body mass index (BMI), hypertension, diabetes, coronary heart disease, six-minute walking test (6MWT), forced expiratory volume in the first second (FEV1), forced vital capacity (FVC), C reactive protein (CRP) before LTx, primary disease, surgical type, intra-operative ECMO type, operation time, post-operative lactic acid (Lac), PaO2/FiO2, PaCO_2_, driving pressure (DP), FiO_2_, peak inspiratory pressure (Pinsp), positive end-expiratory pressure (PEEP), respiratory rate (RR) at T0 (defined within two hours after arrival at the ICU when the patient’s ventilation and hemodynamic condition had stabilized), post-operative ventilator time, ICU stay and 90-day survival after LTx.

### Outcomes

The primary outcome was mechanical ventilation time after LTx. The secondary outcomes were as follows: ICU stay and 90-day survival after LTx.

### Definition

ECMO support for lung transplant recipients is generally decided by a multidisciplinary team of surgeons, intensive care physicians, and pulmonologists according to the donor's condition, the recipient's pre-operative condition, and the intra-operative respiratory and circulatory function status. ECMO weaning in our center precedes ventilator weaning. An ECMO weaning trial was performed according to the 2021 Guideline from the Extracorporeal Life Support Organization (ELSO)^9^.

The extubation criteria were: (1) Spontaneous breathing trial (SBT) ≥ 2 h; (2) No or low-dose vasopressor drug use; (3) Glasgow coma scale score ≥ 13; (4) Upper airway is unobstructed; (5) Improved imaging of chest X-ray; (6) pH > 7.35; (7) Hb > 9 mg/L; (8)Temperature ≤ 38 °C. Extubation failure criteria included requirement of endotracheal intubation within 48 h of extubation.

Measurement method of DP: within 2 h of admission to the ICU, all patients were in supine position without spontaneous respiration. If not, safe doses of sedative, analgesic, and muscle relaxants may be used during measurement to achieve no spontaneous breathing. DP = Plateau Pressure (Pplat-PEEP). After the measurement, proceed to apply the previous ventilator parameter settings.

PMV was defined as mechanical ventilation > 5 days after LTx using ECMO support with or without tracheostomy.

### Statistical analysis

Statistical power calculations were not conducted since the sample size was based on all available data. Using means ± standard deviation or median (interquartile range) to present continuous variables, categorical variables were expressed as numbers (percentages). Student’s t test or the Mann–Whitney *U* test was used to compare continuous variables between the PMV and NPMV groups, as appropriate. Categorical variables were tested by Chi-square test or Fisher’s exact test. For the survival analysis, the effect of PMV was assessed using the log-rank test. The effect of the factors was assessed using univariate and multivariate logistic regressions. Factors with P < 0.1 in univariate logistic regressions were selected for the multivariate analysis. The independent risk factors of PMV were displayed in the nomogram to provide a visual point system to estimate the risk of PMV.

### Performance assessments

Receiver operating characteristic curve (ROC) analysis was performed to assess the predictive ability of the nomogram. The Hosmer–Lemeshow (H–L) goodness-of-fit test was used to evaluate the fit of the model. Bootstrapped calibration curves were used to observe the nomogram performance. The decision-curve analysis (DCA) was used to assess the clinical utility and net benefit. To reduce overfitting, the nomogram was internally validated with the same methods. Statistical analysis was performed using SPSS 25.0 and R version 4.3.0 with the packages of rms, rmda, forestplot, tidyr, dplyr, pROC, survival and ResourceSelection. A two-sided P value < 0.05 was considered as statistically significant.

## Results

### Subject demographics

**Figure 2:**
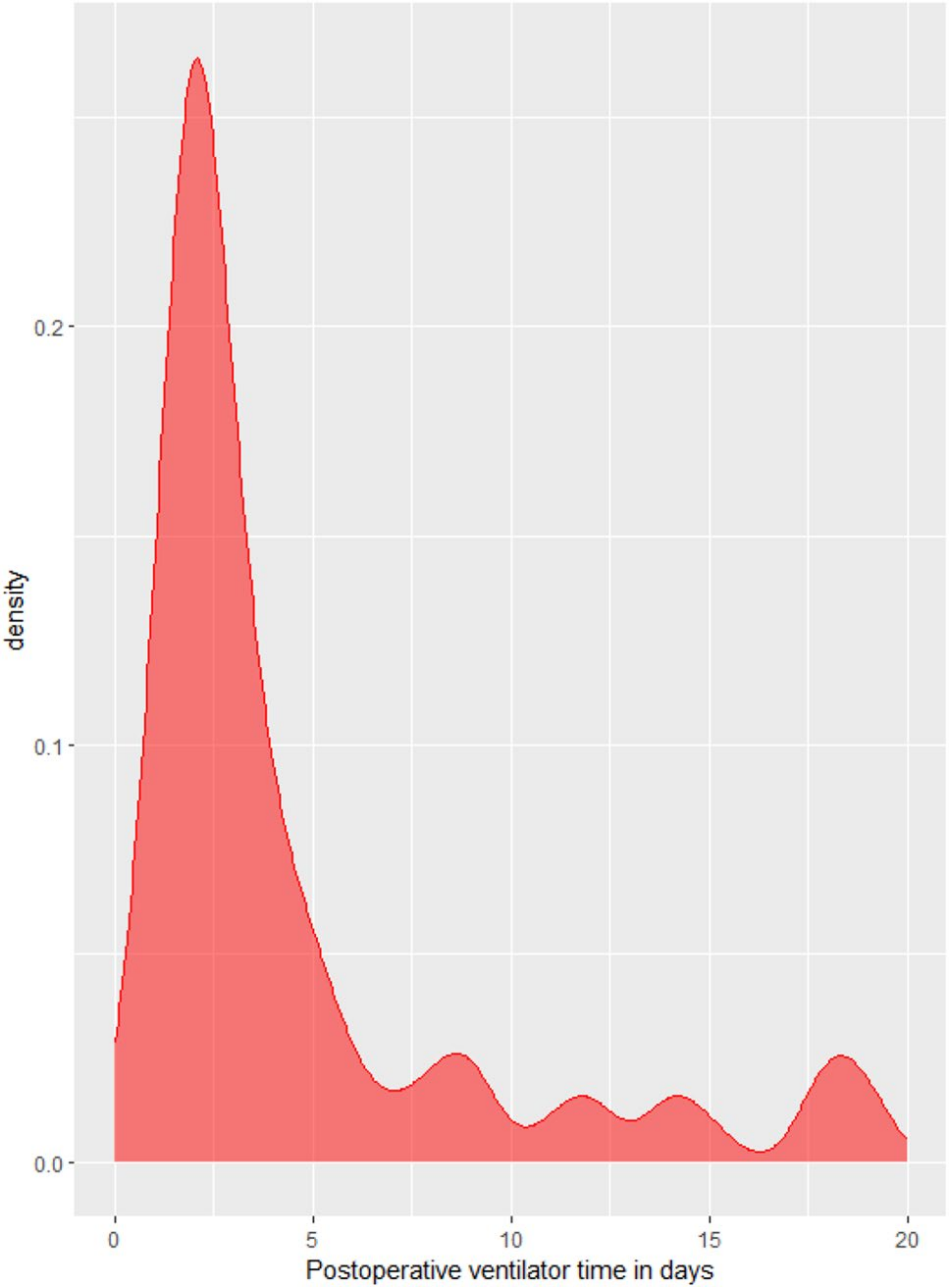
mechanical ventilation time density plot.

Clinical data of 174 patients using ECMO as a bridge to LTx were collected. Two patients with re-transplant/multi-organ transplantation, two with intra-operative use of V-A-V ECMO, and ten incomplete medical records were excluded. As shown in Fig. 1, a total of 174 patients were randomly divided into training set (122) and validation set (52). The training set included 27 patients in PMV group and 95 patients in NPMV group, and the validation set included 11 patients in the PMV group and 41 patients in the NPMV group. A total of mechanical ventilation time density plot is illustrated in Fig. 2. There were no significant differences between training set and validation set (Table 1). In the training set, the most frequent primary disease was idiopathic pulmonary fibrosis (IPF). A total of 56.6% of the patients received bilateral lung transplant, and 43.4% of the patients received unilateral lung transplant. A percentage of 82.0% patients received V–V ECMO, and 18.0% of the patients received V-A ECMO. The median ventilator time was 3 days, and 27 (22.1%) patients underwent PMV.

**Table 1:** Cohort characteristics of patients.

Variables	Training set (n = 122)	Validation set (n = 52)	P value	Training set (n = 122)		P value
				Non-PMV (n = 95)	PMV (n = 27)	
Age (years), mean (SD)	53.1 ± 2.4	53.0 ± 3.5	0.962	53.0 ± 2.5	53.1 ± 4.6	0.957
Male sex, n (%)	94 (77.0)	38 (73.1)	0.891	77(80.9)	17 (63.2)	0.097
BMI, mean (SD)	21.7 ± 1.1	22.1 ± 1.5	0.594	21.0 ± 0.9	22.8 ± 1.8	0.010
Hypertension, n (%)	18 (14.8)	8 (15.4)	0.814	14 (14.7)	4 (14.8)	0.949
Diabetes, n (%)	23 (18.9)	10 (19.2)	0.731	17 (17.9)	6 (22.2)	0.621
Coronary heart disease, n (%)	17 (13.9)	6 (11.5)	0.574	13 (13.7)	4 (14.8)	0.838
6MWT (meter), mean (SD)	211.4 ± 16.2	209.1 ± 17.3	0.655	217.1 ± 18.1	191.0 ± 23.9	0.042
FEV1, mean (SD)	1.00 ± 0.12	1.03 ± 0.10	0.938	1.02 ± 0.09	0.95 ± 0.13	0.457
FVC, mean (SD)	1.47 ± 0.10	1.41 ± 0.15	0.838	1.49 ± 0.10	1.42 ± 0.13	0.495
Preoperative CRP (mg/L), mean (SD)	9.3 ± 3.2	8.7 ± 3.1	0.417	9.9 ± 3.4	7.3 ± 4.2	0.217
Primary disease, n (%)						
IPF	81 (66.4)	31 (59.6)		65 (68.4)	16 (59.3)	
Others	41 (33.6)	21 (40.4)	0.146	30 (31.6)	11 (40.7)	0.359
Surgical type, n (%)						
Unilateral	53 (43.4)	22 (42.3)		45 (47.4)	8 (29.6)	
Bilateral	69 (56.6)	30 (57.7)	0.974	50 (52.6)	19 (70.4)	0.229
Intraoperative ECMO type, n(%)						
V–V	100 (82.0)	45 (86.5)		77 (81.1)	23 (85.2)	
V–A	22 (18.0)	7 (13.5)	0.731	18 (18.9)	4 (14.8)	0.741
Operation time (min), mean (SD)	372.3 ± 22.5	360.2 ± 32.2	0.552	360.3 ± 21.5	415.3 ± 54.6	0.024
Postoperative Lac (mmol/L), mean (SD)	4.2 ± 0.6	3.9 ± 0.9	0.430	3.7 ± 0.4	6.2 ± 2.1	0.001
PaO_2_/FiO_2_, mean (SD)	241.8 ± 27.2	252.3 ± 32.1	0.620	258.0 ± 28.6	181.6 ± 43.3	0.019
PaCO_2_ (cmH_2_O), mean (SD)	36.1 ± 3.2	36.3 ± 2.9	0.952	36.7 ± 2.1	35.4 ± 4.4	0.567
DP (cmH_2_O), mean (SD)	14.3 ± 0.9	14.0 ± 1.1	0.901	13.5 ± 0.8	17.2 ± 2.2	0.001
FiO_2_, mean (SD)	47 ± 1.9	47 ± 2.1	0.911	47 ± 1.6	48 ± 2.8	0.824
Pinsp (cmH_2_O), mean (SD)	20 ± 1.3	21 ± 2.1	0.510	20 ± 0.6	21 ± 1.2	0.607
PEEP (cmH_2_O), mean (SD)	6 ± 0.5	6 ± 0.9	0.722	6 ± 0.3	7 ± 0.8	0.343
RR, mean (SD)	14 ± 0.7	14 ± 2.1	0.833	14 ± 0.4	13 ± 1.1	0.139
Postoperative ventilator time (day), mean (SD)	(2.0, 5.0)	(2.0, 5.0)	0.950	(2.0, 3.0)	(8.0, 18.0)	< 0.001
Postoperative ICU stay (day), median (IQR)	(3.0, 8.0)	(3.0, 9.0)	0.524	(3.0, 5.0)	(6.0, 14.3)	< 0.001
90 day survival, n(%)	91 (74.6)	37 (71.2)	0.519	82 (86.3)	9 (32.3)	< 0.001

*BMI* body mass index, *6MWT* 6 min walking test, *FEV1* Forced Expiratory Volume in the first second, *FVC* forced vital capacity, *CRP* C reactive protein, *IPF* idiopathic pulmonary fibrosis, *COPD* Chronic obstructive pulmonary disease, *ECMO* extracorporeal membrane oxygenation, *Lac* lactic acid, *DP* Driving pressure, *Pinsp* peak inspiratory pressure, *PEEP* positive end-expiratory pressure, *RR* respiratory rate.

### Model development

**Figure 3:**
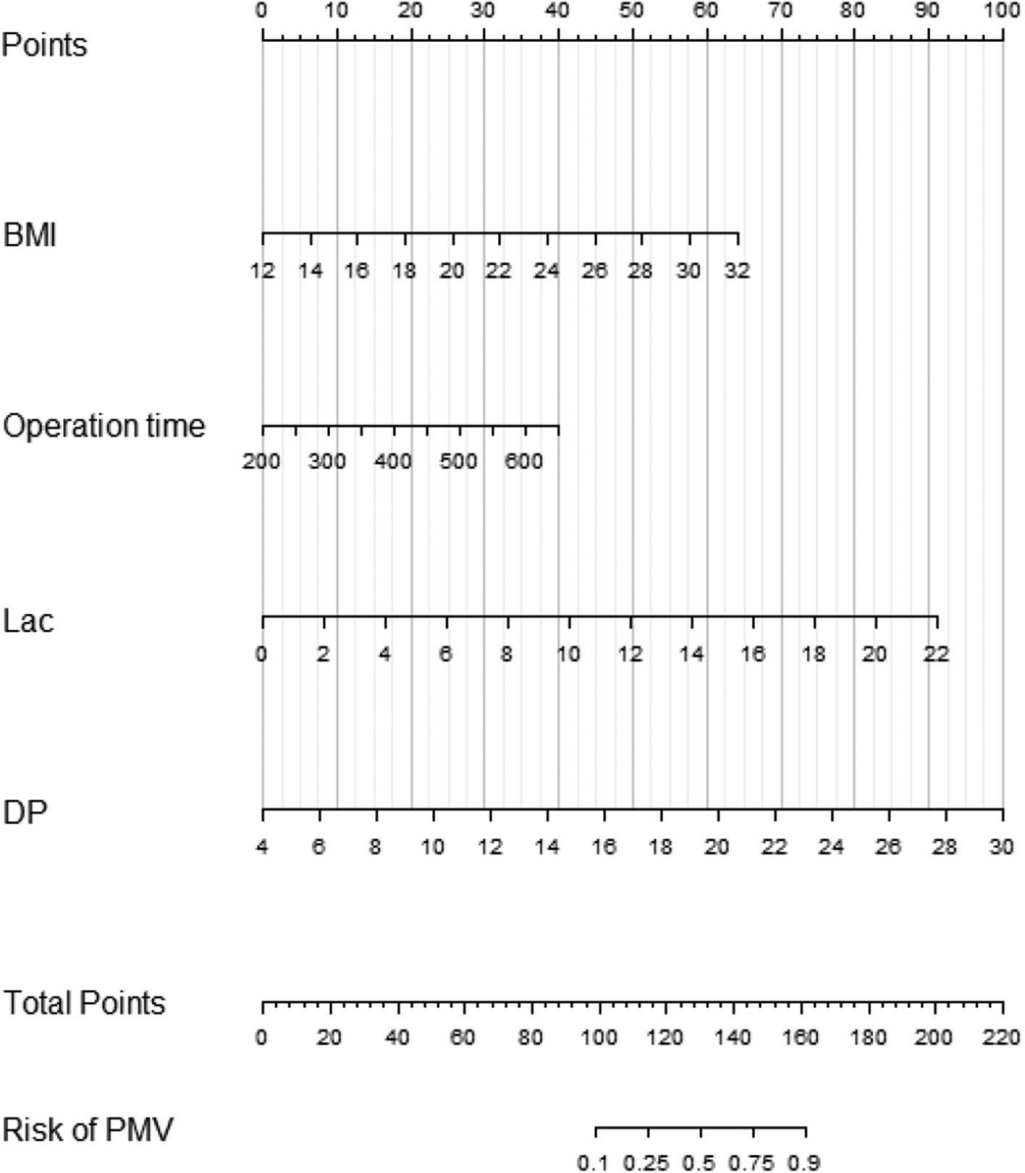
A novel nomogram to predict PMV. The nomogram provides a visual point system based on the combination of patient characteristics (a BMI, operative time, Lac and DP) to estimate the probability of PMV. To calculate the probability of PMV, the points of four variables determined on the scale were added to obtain the total points. Draw a vertical line from the total points scale to the last axis to obtain the corresponding probability of PMV. *BMI* body mass index, *Lac* lactic acid, *DP* Driving pressure.

Univariate analysis identified that six variables were statistically significantly associated with PMV in the training set (Table 2). Multivariate analysis identified that elevated BMI (Odds ratio (OR) 1.252, 95% CI 1.061–1.477, p = 0.008), prolonged operative time (OR 1.006, 95% CI 1.001–1.013, p = 0.048), increased Lac (OR 1.337, 95% CI 1.024–1.745, p = 0.033) and increased DP (OR 1.309, 95% CI 1.099–1.560, p = 0.003) were independent predictors for PMV (Table 2). These four factors were consequently used to develop a nomogram to predict the probability of PMV (Fig. 3).

**Table 2:** Univariable and multivariable logistic analysis.

Variables	Univariable			Multivariable		
	HR	95% CI	P value	HR	95% CI	P value
BMI	1.233	1.023–1.497	0.005	1.252	1.061–1.477	0.008
6MWT, meter	0.993	0.988–0.997	0.043			
Operation time, (min)	1.003	0.998–1.006	0.065	1.006	1.001–1.013	0.048
Lac, (mmol/L)	1.331	1.095–1.684	0.023	1.337	1.024–1.745	0.033
PaO_2_/FiO_2_	0.995	0.993–0.998	0.077			
DP, (cmH_2_O)	1.267	1.083–1.558	< 0.001	1.309	1.099–1.560	0.003

### Model performance and internal validation

**Figure 4:**
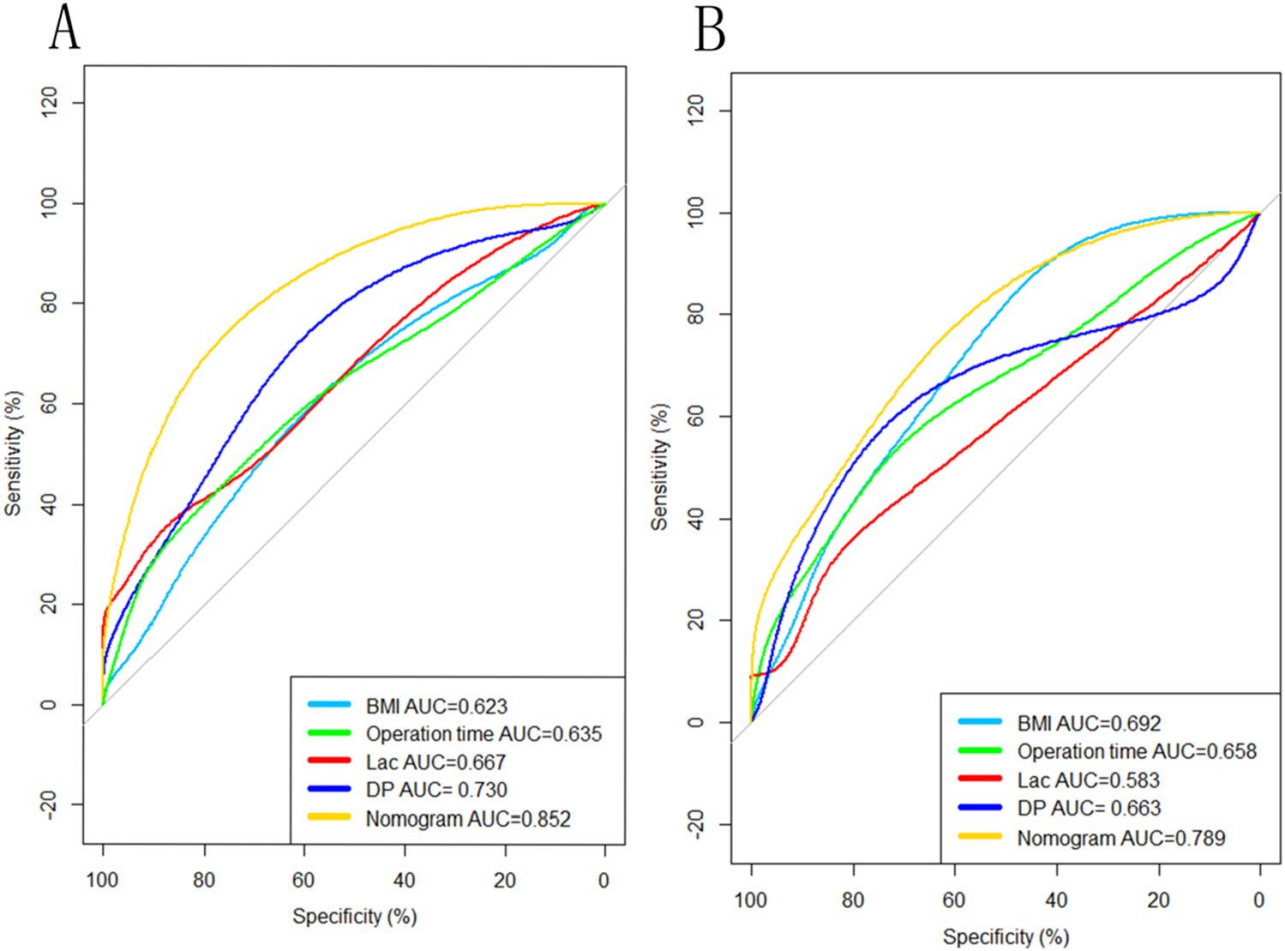
(**A**) ROC analysis for the nomogram of the prediction model and other factors of PMV in Training set. The final integrated model in the figure has an area under the ROC curve of 0.852. (**B**) ROC analysis for the nomogram of the prediction model and other factors of PMV in Validation set. The final integrated model in the figure has an area under the ROC curve of 0.789.

**Figure 5:**
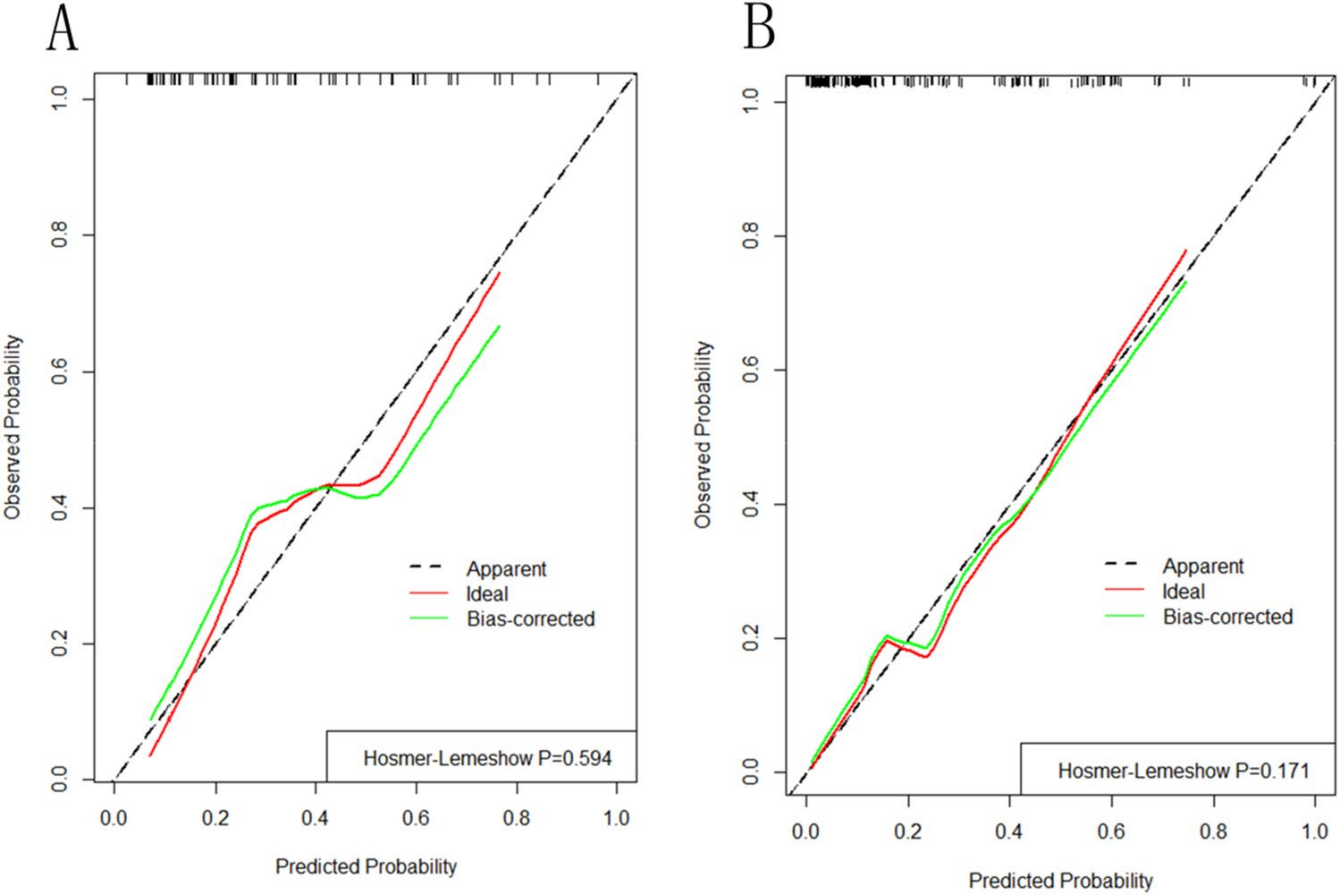
(**A**) A calibration curve of this risk prediction nomogram in Training set. The model calibration has been depicted by bootstrapped calibration curve showing ideal, apparent, and bias-corrected model. (**B**) A calibration curve of this risk prediction nomogram in Validation set. The model calibration has been depicted by bootstrapped calibration curve showing ideal, apparent, and bias-corrected model.

In the training set, the area under the curve (AUC) of the prediction model was 0.852, which was higher than all other factors (Fig. 4A). The H–L goodness-of-fit test value was 0.171. The apparent calibration curve was close to the 45° ideal line, indicating that the observed probability was consistent with the predicted probability (Fig. 5A). Internal validation was additionally performed. The AUC of the prediction model was 0.789 (Fig. 4B). The H–L goodness-of-fit test value was 0.594. The 1000-bootstrap approach was followed, and the bias-corrected calibration curve also demonstrated that the prediction model was well calibrated (Fig. 5B).

### DCA for the prediction model development

**Figure 6:**
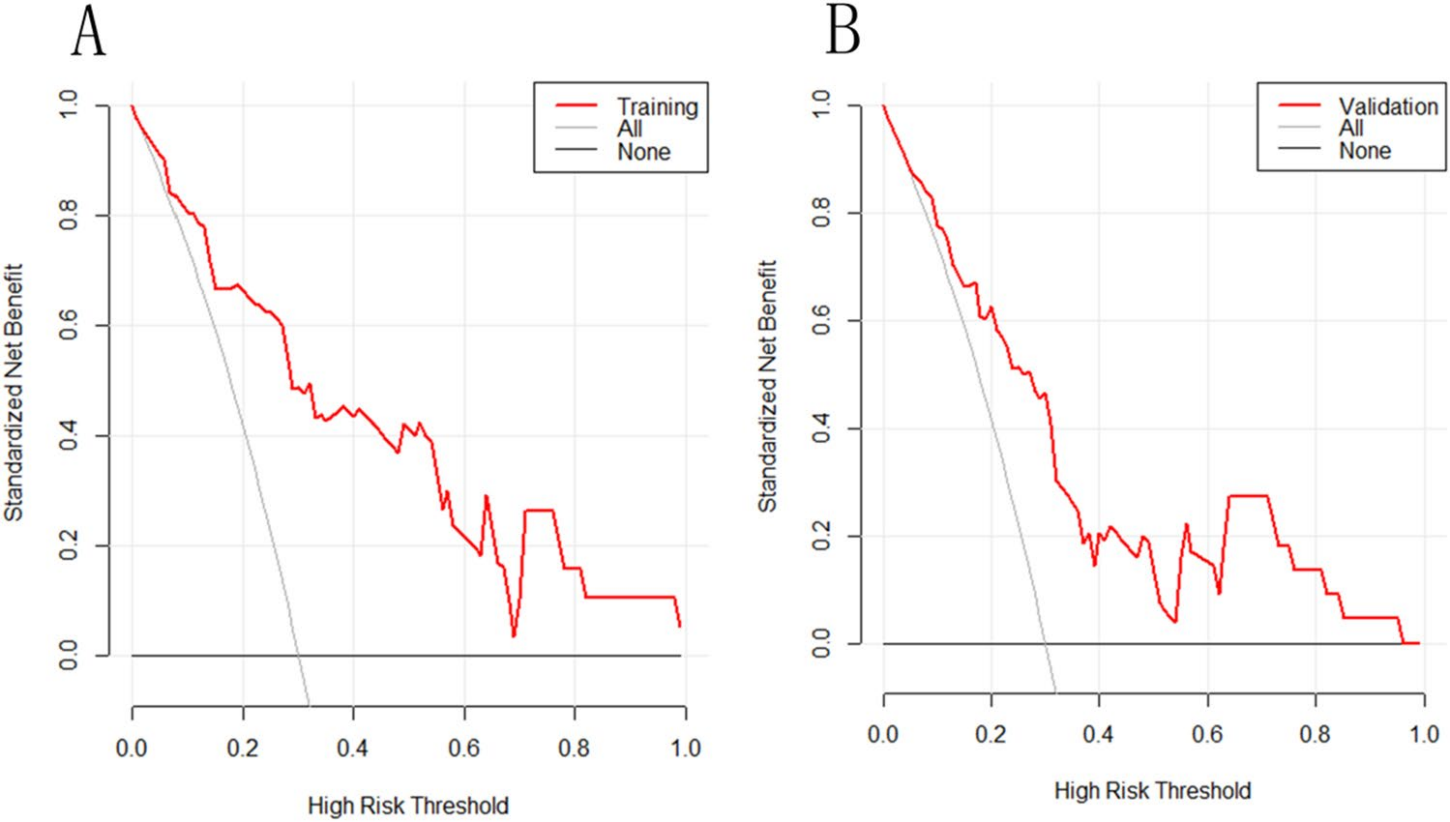
The decision curve analysis (DCA) of the prediction model of recipients with prolonged mechanical ventilation after lung transplantation based on all indicators and all variables. The prediction model or index with the largest net benefit has the best clinical guidance efficiency. Net benefit is defined as the true positive rate minus the weighted false positive rate under a given threshold probability, which defines the high risk of prolonged mechanical ventilation after lung transplantation. (**A**) This DCA could provide a larger net benefit, with ranges of 0% to 100% in Training set. (**B**) This DCA could provide a larger net benefit, with ranges of 0% to 100% in Validation set.

The depicted DCA was used to test whether model-based decisions had clinical applicability compared to the default strategy. Such analyses provide insight into the range of predicted risk for which the model has a higher net benefit than simply either treating all (slope line) patients versus treating no (horizontal line) patient. In other words, a prediction model is only useful at the threshold risk. The graphically depicted DCA indicated the expected net benefit (red curve) per patient for predicting the risk of PMV. In the training and validation sets, within the threshold risk range of 0–100%, intervention decisions based on the predictive model are clearly beneficial (Fig. 6).

### Kaplan–Meier survival curves in all patients

**Figure 7:**
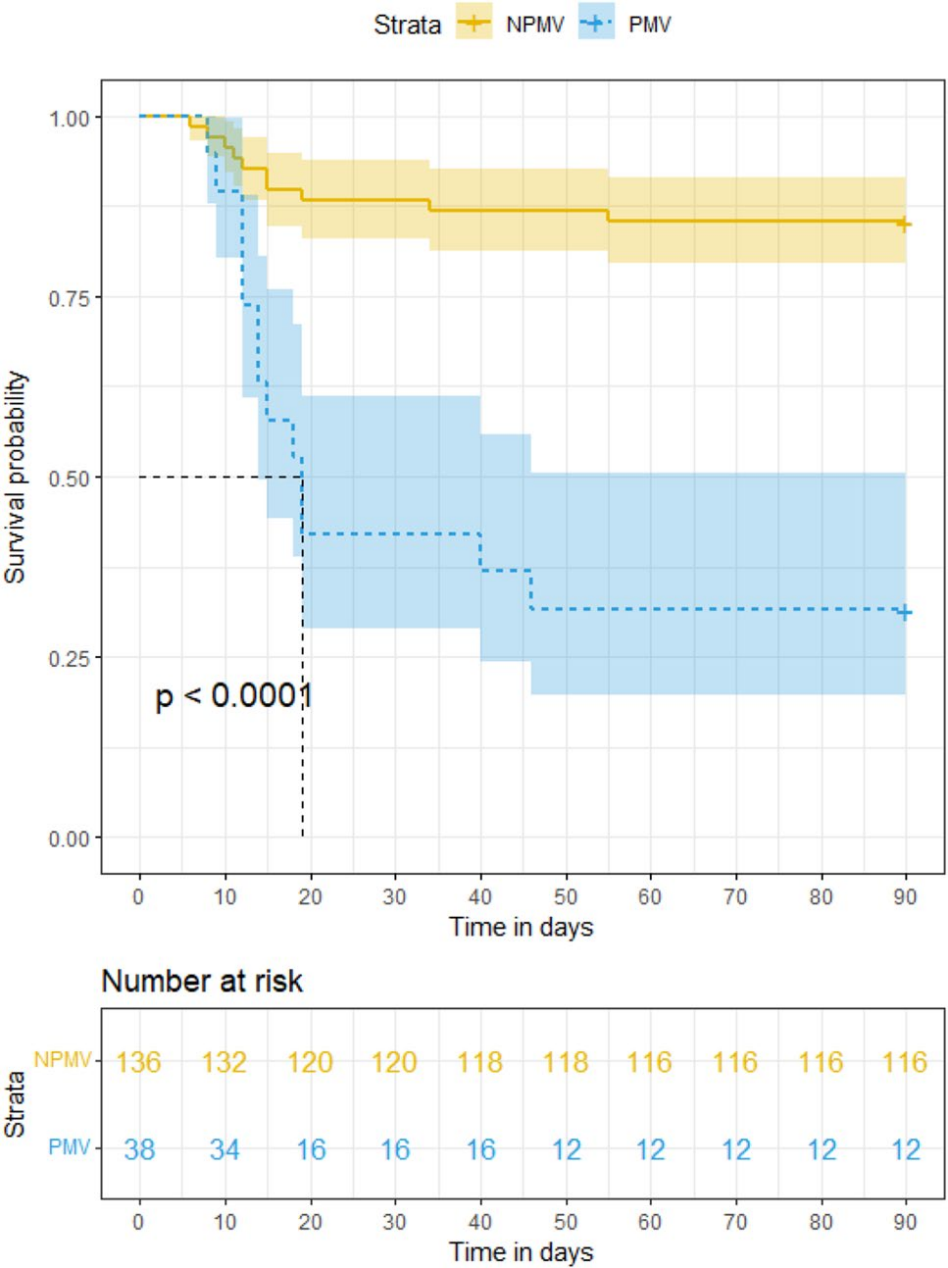
Kaplan–Meier survival curves in patients.

The 90-day mortality rates were 14.7% and 68.4% in the NPMV and PMV groups, respectively. The difference in survival curves in the NPMV and PMV groups was statistically significant (P < 0.001, Fig. 7).

## Discussion

The occurrence of PMV in in-patients using ECMO as a bridge to LTx was 21.8% in the present study. Gao et al. reported that the incidence of PMV (defined as > 72 h) was 31.9%, excluding ECMO-supportive patients^10^. Since PMV is not clearly defined in the literature, the PMV in this study was defined according to the previous large-scale retrospective study, where the mean ventilation time of LTx patients was 5 days^11^. Therefore, PMV was defined as > 5 days in the current research. The median ventilation time was 12 days in the PMV group and 2 days in the NPMV group. The 90-day survival in the PMV group was significantly reduced in the K-M analysis curve. LTx patients supported by ECMO had poor lung function and 6MWT results. ECMO was used continuously during and after LTx to improve the hemodynamic state, facilitate gas exchange and provide support for the acute phase of severe PGD^12^. The present investigation suggests that ECMO type was not a risk factor for PMV and that appropriate use of V–V ECMO and V–A ECMO did not affect ventilation time in general. Four independent predictors of PMV were hereby used to create a novel nomogram to estimate the decision to provide PMV with good AUC and calibration in internal validation, including BMI, operative time, Lac and DP.

Patients with an elevated BMI have an increased risk of chronic diseases (diabetes, hypertension, dyslipidemia, etc.), impaired intra-operative visibility, and an increased risk and duration of surgery^13^. Moreover, changes in respiratory mechanics, such as static compliance of the chest wall changes and upper airway resistance increases, result in atelectasis and increased post-operative complications^14^. Previous studies have reported that recipient obesity was an independent risk factor for PGD3 and was also associated with lower 90-day survival^15^.

Prolonged operative time may affect the following aspects: prolonged cold ischemia of the donor lung may cause increased graft rejection^16^; prolonged anesthesia increases the risk of respiratory muscle weakness and slower recovery of anesthesia^17^; there is an increased chance of infection of the lungs with mechanical ventilation during surgery^18^. Intraoperative use of ECMO has several advantages including achieve lung-protective ventilation strategy, effectively reduce pulmonary blood flow, and provide hemodynamic stabilization. The study found prophylactic postoperative ECMO prolongation was associated with excellent outcomes in patients with pulmonary hypertension and questionable graft function at the end of implantation. However, Intraoperative and postoperative ECMO prolongation can be associated with worse outcomes such as bleeding complications, systemic inflammatory response, acute kidney injury, and thromboembolic complications^19,20^, There is no consensus on the Intraoperative ECMO prolongation. The prolongation of the operation time reflects the high difficulty of the operation, which passively prolongs the application time of ECMO^21^. In summary, it affects the prognosis of LTx patients.

Imbalance between oxygen supply and oxygen consumption is an important factor in post-operative hyperlactatemia^22^. Although the application of ECMO can increase oxygen supply, the contact between blood and biological materials leads to the activation of white blood cells and endothelial cells, body temperature changes, or intestinal endotoxin release, which can cause systemic inflammatory response syndrome, and lead to an increase in cellular oxygen consumption. In addition, intra-operative blood loss will reduce the ability of blood to deliver oxygen, and body fluid loss will also bring a decrease in effective blood volume, which in turn increases the risk of tissue and organ hypoperfusion. Additionally, hemodilution, non-pulsatile perfusion and other factors can influence the microcirculation of important tissues and organs. These reasons lead to a significant increase in lactic acid concentration^23^. Severe hyperlactic acidemia decreases myocardial contractility and cardiac output, and also reduces the cardiovascular response to vasoactive drugs, further affecting tissue perfusion. Therefore, intra-operative lactate monitoring should be strengthened, because it is necessary to reduce lactate levels through fluid resuscitation, maintain reasonable blood pressure, and appropriate increase in blood and gas flow in ECMO, which may improve circulatory function, facilitate the recovery process and reduce the occurrence of PMV.

DP is the most significant risk factor for PMV in ventilator parameters, which can reflect respiratory compliance. DP was first introduced by Amato et al. in 2015 in their meta-analysis study for acute respiratory distress syndrome (ARDS) patients^24^. They reported that high DP was most strongly associated with worse survival. PGD is an acute lung injury syndrome that occurs in the first 72 h after LTx^25^ and is characterized by pulmonary edema with diffuse alveolar damage. Clinical manifestations are progressive hypoxemia and radiographic pulmonary infiltrates without other identifiable causes. PGD is similar to ARDS in many ways. PGD3 at T0 in previous studies was a risk factor for PMV. The grade of PGD is based on PaO_2_/FiO_2_ and X-ray^26^. After ECMO application, PaO_2_/FiO_2_ does not fully reflect lung function, and turning off ECMO gas source measurement may cause irreversible damage. PGD grade was not collected in this study. Further research is needed on the association between DP and PGD grade in patients using ECMO as a bridge to LTx.

The present study has several limitations. First, it single-center study, However, hundreds of ECMO-supported lung transplants are performed every year in our center, with appropriate management experience. Due to the lack of large-scale LTx centers in China, external verification has not been completed. Second, the sample size was relatively small. Finally, this study did not include donor information, intra-operative anesthesia, and ECMO parameter settings, which may reduce the predictive effect of nomograms.

## Conclusions

Through retrospective analysis of patients using extracorporeal membrane oxygenation as a bridge to lung transplantation, a new nomogram for predicting the risk of prolonged mechanical ventilation was hereby established with satisfactory internal validation performance. Optimizing four factors including body mass index, operative time, lactic acid at T0 and driving pressure may reduce the risk of prolonged mechanical ventilation.

## Data Availability

The datasets used and analysed during the current study are available from the corresponding author on reasonable request.
